# Supplementary material for: Impact of built environment on residential online car-hailing trips: Based on MGWR model
Source: PLoS One. 2022 Nov 17;17(11):e0277776. doi: 10.1371/journal.pone.0277776 (PMC9671434; doi:10.1371/journal.pone.0277776)
Supplement: S1 Table — (DOCX) [file pone.0277776.s001.docx]

**S1 Table. Spatial autocorrelation.**

|  | Moran's Index | Expected Index | Variance | Z-score | P-value |
| --- | --- | --- | --- | --- | --- |
| Restaurant | 0.360574 | -0.00022 | 0.000109 | 34.58997 | 0 |
| Shopping | 0.136351 | -0.00022 | 0.000105 | 13.34576 | 0 |
| Living service | 0.384635 | -0.00022 | 0.000108 | 36.96689 | 0 |
| Corporation | 0.297133 | -0.00022 | 0.000108 | 28.67689 | 0 |
| Financial service | 0.276597 | -0.00022 | 0.000108 | 26.58921 | 0 |
| Education & culture | 0.325588 | -0.00022 | 0.000108 | 31.30262 | 0 |
| Business residence | 0.591032 | -0.00022 | 0.000109 | 56.67351 | 0 |
| Entertainment | 0.274528 | -0.00022 | 0.000108 | 26.4161 | 0 |
| Medical facility | 0.346654 | -0.00022 | 0.000108 | 33.40126 | 0 |
| Governmental agency | 0.211360 | -0.00022 | 0.000108 | 20.32123 | 0 |
| Scenic spot | 0.254438 | -0.00022 | 0.000097 | 25.82979 | 0 |
| Accommodation | 0.339249 | -0.00022 | 0.000096 | 34.63801 | 0 |
| Internal traffic | 0.038498 | -0.00022 | 0.000109 | 3.709374 | 0.000208 |
| Road | 0.358406 | -0.00022 | 0.000109 | 34.3704 | 0 |
| Population | 0.696790 | -0.00022 | 0.000107 | 67.24012 | 0 |
| Transportation hub | 0.545849 | -0.00022 | 0.000105 | 53.26673 | 0 |
| HHI | 0.160754 | -0.00022 | 0.000109 | 15.42172 | 0 |
| Housing price | 0.915183 | -0.00022 | 0.000109 | 87.71724 | 0 |
