# Supplementary material for: Impact of built environment on residential online car-hailing trips: Based on MGWR model
Source: PLoS One. 2022 Nov 17;17(11):e0277776. doi: 10.1371/journal.pone.0277776 (PMC9671434; doi:10.1371/journal.pone.0277776)
Supplement: S2 Table — (DOCX) [file pone.0277776.s002.docx]

**S2 Table. Correlation coefficient test table for each variable.**

|  | RT | SP | LS | CP | FS | EC | BR | ET | MF | GA | SS | AC | IT | RD | PU | HHI | TH | HP |
| --- | --- | --- | --- | --- | --- | --- | --- | --- | --- | --- | --- | --- | --- | --- | --- | --- | --- | --- |
| RT | 1 |  |  |  |  |  |  |  |  |  |  |  |  |  |  |  |  |  |
| SP | .370^**^ | 1 |  |  |  |  |  |  |  |  |  |  |  |  |  |  |  |  |
| LS | .639^**^ | .379^**^ | 1 |  |  |  |  |  |  |  |  |  |  |  |  |  |  |  |
| CP | .211^**^ | .139^**^ | .209^**^ | 1 |  |  |  |  |  |  |  |  |  |  |  |  |  |  |
| FS | .305^**^ | .260^**^ | .318^**^ | .537^**^ | 1 |  |  |  |  |  |  |  |  |  |  |  |  |  |
| EC | .334^**^ | .222^**^ | .354^**^ | .446^**^ | .416^**^ | 1 |  |  |  |  |  |  |  |  |  |  |  |  |
| BR | .480^**^ | .239^**^ | .440^**^ | .298^**^ | .331^**^ | .358^**^ | 1 |  |  |  |  |  |  |  |  |  |  |  |
| ET | .487^**^ | .311^**^ | .444^**^ | .235^**^ | .334^**^ | .443^**^ | .283^**^ | 1 |  |  |  |  |  |  |  |  |  |  |
| MF | .365^**^ | .216^**^ | .403^**^ | .093^**^ | .218^**^ | .215^**^ | .339^**^ | .182^**^ | 1 |  |  |  |  |  |  |  |  |  |
| GA | .201^**^ | .086^**^ | .174^**^ | .222^**^ | .201^**^ | .297^**^ | .386^**^ | .164^**^ | .186^**^ | 1 |  |  |  |  |  |  |  |  |
| SS | .085^**^ | 0.022 | .035^*^ | .041^**^ | .052^**^ | .080^**^ | .069^**^ | .075^**^ | .049^**^ | .093^**^ | 1 |  |  |  |  |  |  |  |
| AC | .239^**^ | .158^**^ | .208^**^ | .150^**^ | .209^**^ | .202^**^ | .270^**^ | .231^**^ | .088^**^ | .108^**^ | .033^*^ | 1 |  |  |  |  |  |  |
| IT | .200^**^ | .138^**^ | .180^**^ | .075^**^ | .163^**^ | .119^**^ | .129^**^ | .126^**^ | .154^**^ | .088^**^ | 0.014 | .059^**^ | 1 |  |  |  |  |  |
| RD | .158^**^ | .122^**^ | .116^**^ | .177^**^ | .215^**^ | .165^**^ | .117^**^ | .146^**^ | .121^**^ | .081^**^ | .073^**^ | .079^**^ | .238^**^ | 1 |  |  |  |  |
| PU | .030^*^ | 0.015 | 0.013 | .034^*^ | .038^**^ | .071^**^ | .052^**^ | 0.014 | 0.007 | .075^**^ | 0.011 | -0.001 | -0.025 | 0.001 | 1 |  |  |  |
| HHI | .061^**^ | .035^*^ | .073^**^ | .119^**^ | .147^**^ | .110^**^ | .083^**^ | .061^**^ | .065^**^ | .041^**^ | 0.019 | .081^**^ | .048^**^ | .119^**^ | -0.019 | 1 |  |  |
| TH | -0.001 | 0.020 | -0.027 | 0.004 | -0.013 | -0.018 | -0.022 | -0.019 | -0.009 | 0.000 | -0.007 | 0.021 | 0.017 | .044^**^ | -0.028 | .175^**^ | 1 |  |
| HP | -.029^*^ | 0.006 | -.049^**^ | -0.023 | .082^**^ | .065^**^ | -0.009 | .068^**^ | -.044^**^ | 0.006 | .051^**^ | .094^**^ | .051^**^ | .111^**^ | 0.013 | .070^**^ | 0.004 | 1 |

Note:

RT: Restaurant; SP: Shopping; LS: Living house; CP: Corption; FS: Financial service; EC: Education & culture; BR: Business residence; ET: Enterment; MF: Medical facility; GA: Government agency; SS: Science spot; AC: Accomdation; IT: Internal traffic; RD: Road; PU:Population; TH: Transportation hub; HP: Housing price.

** Significant at 0.01 level; * Significant at 0.05 level
