# Supplementary material for: Impact of built environment on residential online car-hailing trips: Based on MGWR model
Source: PLoS One. 2022 Nov 17;17(11):e0277776. doi: 10.1371/journal.pone.0277776 (PMC9671434; doi:10.1371/journal.pone.0277776)
Supplement: Attachment — Submitted filename: Response to Reviewers.docx [file pone.0277776.s003.docx]

Dear Editors and Reviewers:

Thank you for your letter and for the reviewers’ comments concerning our manuscript entitled “Analysis of urban residents' online car-hailing journey characteristics based on the MGWR model” (ID: PONE-D-22-20651). Those comments are all valuable and very helpful for revising and improving our paper, as well as the important guiding significance to our researches. We have studied comments carefully and have made correction which we hope meet with approval. Revised portion are marked in red in the paper. The main corrections in the paper and the responds to the reviewer’s comments are as fllowing.

We sincerely hope the new version meet with the publication standard of plos one. Please let us know if anything else is needed and we look forward to hearing from you soon.Thank you and best regards.

Yours sincerely,

Yan Cao

Corresponding Author:

Yongzhong Tian

Associate Professor

School of Geographical Sciences, Southwest University

Chongqing 400715, China

Tel/Fax: +86-17729619166

Email: tyzlf@swu.edu.cn

**Responds to the reviewer #1’s comments**

**Point 1:** *The paper is poorly expressed in language. Pay attention to the mixed use of past and present tense, especially, in the same sentence.*

**Response:** We are very sorry that our incorrect writing has caused you problems in reading. Our manuscripts have been edited by English language editors before submission, but due to our negligence there may still be some errors in the text. We have carefully checked English throughout the manuscript and made a comprehensive revision in the new manuscript.

**Point 2:**  *There are too many abbreviations in the paper, which seriously affects the reading as well as the understanding of the contents.*

**Response:** We are so sorry that the abbreviations in the manuscript have caused confusion in your reading. Unnecessary abbreviations in the manuscript were reduced and we retained the abbreviations of the study periods, such as L227-L228.

**Point 3:** *The introduction needs to be reorganized to highlight the value and meaning of the research. Too much discussion of variables selection is not appropriate for the introduction.*

**Response:** We reorganized the introduction and divided it into Introduction and Literature review. In the introduction, we emphasized the value and significance of this study, and summarized previous studies on variable selection and influence model in the literature review.

**Point 4:** *The discussion of the results is mainly on the sign of the variables, and the analysis of the causes of the effects is not in-depth. The number of variables of interest are too much, resulting in unfocused findings.*

**Response:** Thank you for your valuable comments. The results section were reorganized. We analysed the spatial and temporal characteristics of residential trips, then compared the main variables in the results of each model and explored the causes, and in the discussion section the key variables that passed all the model tests were analysed.

**Point 5:** *The conclusion is brief and the policy recommendations lack relevance and application.*

**Response:** We have expanded the concluding section. The novelty and value of the findings of this study were emphasised, and each of the findings in our research process was analysed for its applicability.

**Point 6:** *The title is not specific enough and does not indicate the core contents of the study. The introduction section points out "the purpose of this study is to examine the spatiotemporal relationship between online cab-hailing and the urban built environment", but the title is a study of "characteristics", which is confusing. It is recommended to revise it to cover and reflect your core research contents.*

**Response:** It is really true as Reviewer suggested that the title does not cover and reflect our core research contents. We thought carefully about the content of our title and revised it, changing the original title to " Impact of built environment on residential online car-hailing trips: based on MGWR model ".

**Point 7:** *Unify the expressions of the core terminology in the whole paper.
(1) Such as: car-hailing or cab-hailing? Please explain to me the difference between the two concepts and why you chose this one and not the other? If the two concepts refer to the same content, please unify the expressions in the text directly.
(2) DiDi has an English version of its website, please check the English spelling of DiDi. Please unify the expressions in the whole text.*

**Response:** We are very sorry for neglecting to unify the expressions of the core terminology in the whole paper.

(1) Car-hailing, cab-hailing or ride-hailing have the same concept. The overarching idea of them is that a customer hires a driver to take them exactly where they need to go, something accomplished by hailing a taxi from the street, calling up a car service on the phone,or virtually hailing a car and driver from an app. We unified our expressions and car-hailing was used in the manuscript.
(2) We checked and confirmed their correct English writing of DiDi on DiDi's official website. DiDi was used in the whole text.

**Point 8:** *L67: What are the others referring to?*

**Response:** Thank you very much for your valuable comments. The 5D's principles of built environment and taxi trips were developed by Cervero and Ewing in 1997 and 2010 respectively. We have corrected our writing errors and removed the phrase “as well as others”.

**Point 9:** *L141-L147:
(1) There is actually another peak(13:00-14:00) in Figure 2, why is this not discussed?.
(2) MP and EP? In the case of abbreviations for the first time, please use the whole words.
(3) The presentation of the content is illogical, and the English expression level is very poor.Please have your paper copy edited by a native speaker. Pay attention to the mixed use of past and present tense, especially, in the same sentence. Be consistent.*

**Response:** We have re-written the part:

(1) In the corrected paragraph, we have described the peaks for this time period (13:00-14:00) and the reasons for choosing the morning and evening peaks and late night as the study periods in the manuscript.
(2) Abbreviations have been correcteded in the text.
(3) We have reorganized this section and carefully corrected tense errors.

**Point 10:** *Section 2.2 contains too much contents and the logic is not clear enough. Please rearrange the logic of Section 2 and divide it into a proper chapters.*

**Response:** Considering the Reviewer’s suggestion,we have rearranged the Section 2. We divided the section 2 into " Online car-hailing trips data " and " Built environment variables ". We put it in the section "Study Area" about the content of the research grid division.

**Point 11:**  **L229:** *(1)Where are the Tables A1 and A2? (2)Poor English proficiency and confusing use of tenses in this paragraph.*

**Response:**

(1) Tables A1 and A2 are actually S1 and S2 Tables, they are placed at the end of the manuscript.

(2)We are so sorry for our poor English writing. The tense errors have been carefully corrected.

**Point 12:** *L237~L248: (1)Lack of standardization in table designing. (2)Please correspond to Table 1 for the order of variables in Table 2. (3)What is the difference between R-square and R2 in Table 2? (4)L241: EP? OR EPP? (5)It is not appropriate to discuss the conclusions of Table 4 here. (6)Please explain the meaning of AICc in the note part of Table 2. (7)Please explain on what software or algorithm these results are based?*

**Response:**

(1) All tables in the text have been redesigned to meet the requirements of the journal.

(2) Thanks for your comments. We have reordered the variables in Table 2.

(3) We are very sorry about the use of the wrong abbreviation. R-square is the residual sum of squares in previous versions. In the revised manuscript, we update it to RSS to avoid misunderstandings.

(4) In this place it mainly refers to the EPP model, which has been rewritten in the text.

(5) We have reformatted this section and removed inappropriate parts of it.

(6) Thank you for your sincere and careful comments. We have explained the meaning of AICc in the note part of Table 2.

(7) The calculations of the MGWR model in this paper are based on the Spatial Analysis Research Center (SPARC) at Arizona State University, USA. The MGWR2.2 software is developed by SPARC (<https://sgsup.asu.edu/SPARC>), the maps are produced using ArcGIS 10.6 software. This section has been added to the text in lines 319 to 321.

**Point 13:** *Some of the details:
L124: I don't think it's appropriate for the contents to appear in this sentence: "rich and diverse internal land use variables (variables?)".
Line126: I don't think the contens of the brackets, (such as Jinniu and Chenghua), make sense.
L140, L147: A period is missing at the end of the sentence.
L159: [Please check that I have understood this section correctly.]?
L194: distributions
L197: Table1, the explained variables are usually placed before the explanatory variables.
The Table 4 and Table 5 are misplaced. When discussing tables, please place the table below the text.
Conclusion: Please note the use of punctuation in the whole test.*

**Response:** It is really true as Reviewer suggested that there are some of the details of the errors that occurred during the writing process.

(1) L124: Thank you for your kind comments. We have changed the “variables”to “types”.
(2) L126: We have rewritten the sentence and listed all the study areas covered in our study area.
(3) L140, L194, L197: We have carefully corrected punctuation errors and grammatical errors in our full text.
(4) L159: We are very sorry for our negligence. The irrelevant sentence in the new manuscript have been deleted.
(5) L197: The order of explanatory variables and explained variables were reorganized in Table 1. Considering your valuable suggestion, we have repositioned Tables 4 and 5 and placed them below the corresponding text.

We tried our best to improve the manuscript and made some changes in the manuscript. These changes will not influence the main content and framework of the paper. And here we did not list the changes but marked in red in revised paper.We appreciate for your warm work earnestly, and hope that the correction will meet with approval.
Once again, thank you very much for your comments and suggestions.

**Responds to the reviewer #2’s comments**

**Point 1:** *The manuscript needs language, grammar, and syntactic editing. The English language usage should be checked by a fluent English speaker.*

**Response:** We are very sorry that our incorrect writing has caused you problems in reading. Our manuscripts have been edited by English language editors before submission, but due to our negligence there may still be some errors in the text. We have carefully checked English throughout the manuscript and made a comprehensive revision in the new manuscript.

**Point 2:** *The more suitable title should be selected for the article*

**Response**: It is really true as Reviewer suggested that the title does not cover and reflect our core research contents. We thought carefully about the content of our title and revised it, changing the original title to " Impact of built environment on residential online car-hailing trips: based on MGWR model ".

**Point 3:** *Also, find some new related references which should add to the 1st and 2nd parts.*

**Response**: Thank you for your valuable comments. Some new related references were added in the Introduction and Literature review.

**Point 4:** *Must add much more explanations and interpretations for the results, which are not enough. It is suggested to compare the results of the present research with some similar studies which is done before (more justification is needed).*

**Response:** We have made correction according to your valuable comments. The results section were reorganized. We analysed the spatial and temporal characteristics of residential trips, then compared the main variables in the results of each model and explored the causes, and in the discussion section the key variables that passed all the model tests were analysed.

**Point 5:** *Please make sure your conclusions section underscores the scientific value-added of your paper and the applicability of your findings/results, as indicated previously. Please revise your conclusion part into more detail. It would be best if you enhanced your contributions, limitations, underscore the scientific value-added of your paper, and the applicability of your findings/results and future study in this session.*

**Response:** We have re-written this part according to your thoughtful and sincere comments. The novelty and value of the findings of this study were emphasised, and each of the findings in our research process was analysed for its applicability. To illustrate the limitations of this study and future work, we added the " Limitations and future work " section.

We tried our best to improve the manuscript and made some changes in the manuscript. These changes will not influence the main content and framework of the paper. And here we did not list the changes but marked in red in revised paper.We appreciate for your warm work earnestly, and hope that the correction will meet with approval.
Once again, thank you very much for your comments and suggestions.
