# Supplementary material for: Impact of built environment on residential online car-hailing trips: Based on MGWR model
Source: PLoS One. 2022 Nov 17;17(11):e0277776. doi: 10.1371/journal.pone.0277776 (PMC9671434; doi:10.1371/journal.pone.0277776)
Supplement: Attachment — Submitted filename: Comments-round2.docx [file pone.0277776.s004.docx]

The paper has been greatly improved, both in terms of language and logic of expression. The authors' efforts regarding the revision of the second edition are worthy of recognition. In order to improve the quality of the paper, I would like to make the following comments:

1. L39-L47: The discussion of the advantages of DiDi data should be placed in the literature review section. I suggest putting it after L133, the discussion on data limitations. Start a separate paragraph.
2. In the introduction section, three difficulties are presented (L48-L63) and this research has been improved in three ways (L64-L76). Please reorganize the presentation of this part so that the research difficulties and your contribution are presented separately and **correspond to** each other.
3. L64-L65: This is the content of your research, not the purpose. You can directly indicate in the opening section that the contribution of this paper is reflected in the following three areas.
4. L73-L76: A discussion of the model results would be inappropriate here.
5. L76-L80: This is the purpose of your research, please state it on a separate line. Add your content chapter arrangement in this part.
6. L134-L139: The expression is fragmented and not well connected to the literature. Please integrate this section with the "contribution" in the introduction section and highlight your innovation in this section.
7. L239："MGWR uses different bandwidths for each variable, unlike the classical GWR model, which requires different levels of spatial smoothing for each variable;" I think what you are trying to convey is that MGWR can “requires different levels of spatial smoothing for each variable”, but the subject of the second half of this sentence is GWR. Please verify if the meaning of this sentence matches what you are trying to express.
8. It is best not to have a figure in the discussion section about the results. Please put Figure 5 and related contents in another appropriate place. It is suggested that Chapter 3 be discussed in several subsections, and the discussion on Figure 5 could be a subsection of Chapter 3.

Other comments:

9. ! Please be responsible for your own paper, read it carefully and correct all details of the mistakes.

(1) L46, L318: A period is missing at the end of the sentence.

(2) The content of the form should be standardized: Note that the number of decimal points in the tables is the same for the same category of data, so that it doesn't look cluttered.

(3) Make sure that the first letter of the same content is the same in different tables.

(4) Please unify the citation of all references, such as L215, etc.

(5) L226-L228: The letters of the formula and the explanation should correspond in case. j is a lowercase letter.

(6) Please check the text for all grammatical errors. E.g. L267: S1 and S2 Tables show …..; L271: shows?

10. L123: “Second,…..”
11. L149: Two “study area” in one sentence is wordy. The former emphasizes the object of study and the latter emphasizes the area.
12. It is not necessary to emphasize "in this paper” or “in this study". E.g. L163 and L166.
13. L168: too many “and”
14. Use "(1) Variable 1." or other tag before each variable explanation so that it corresponds to your variables selections in the first paragraph.
15. L235-L237: It should be placed before table1.
16. In order to make it easier for readers to distinguish, I suggest writing “Morning peak 1 model (pick-ups), Morning peak 2 model (drop-offs) ’’in Table 2, so as in Table 3-5.
