# Supplementary material for: Impact of built environment on residential online car-hailing trips: Based on MGWR model
Source: PLoS One. 2022 Nov 17;17(11):e0277776. doi: 10.1371/journal.pone.0277776 (PMC9671434; doi:10.1371/journal.pone.0277776)
Supplement: Attachment — Submitted filename: Response to Reviewers.docx [file pone.0277776.s005.docx]

Dear Editors and Reviewers:

Thank you for your letter and for the reviewers’ comments concerning our manuscript entitled “Impact of built environment on residential online car-hailing trips: based on MGWR model” (ID: PONE-D-22-20651R1). Those comments are all valuable and very helpful for revising and improving our paper, as well as the important guiding significance to our researches. We have studied comments carefully and have made correction which we hope meet with approval. Revised portion are marked in red and blue in the paper. The main corrections in the paper and the responds to the reviewer’s comments are as fllowing.

We sincerely hope the new version meet with the publication standard of plos one. Please let us know if anything else is needed and we look forward to hearing from you soon.Thank you and best regards.

Yours sincerely,

Yan Cao

Corresponding Author:

Yongzhong Tian

Associate Professor

School of Geographical Sciences, Southwest University

Chongqing 400715, China

Tel/Fax: +86-17729619166

Email: tyzlf@swu.edu.cn

**Responds to the reviewer #1’s comments**

**Point 1:** *L39-L47: The discussion of the advantages of DiDi data should be placed in the literature review section. I suggest putting it after L133, the discussion on data limitations. Start a separate paragraph.*

**Response**: Thank you for your valuable comments. We reorganized the advantages of DiDi data and put it in the Literature review.

**Point 2:** *In the introduction section, three difficulties are presented (L48-L63) and this research has been improved in three ways (L64-L76). Please reorganize the presentation of this part so that the research difficulties and your contribution are presented separately and correspond to each other.*

**Response:** This section has been reorganized. We have separated the difficulties and improvements so that they correspond to each other.

**Point 3:** *L64-L65: This is the content of your research, not the purpose. You can directly indicate in the opening section that the contribution of this paper is reflected in the following three areas.*

**Response:** Thank you for your sincere opinion. We modified this part of the statement and pointed out our contribution at the beginning.

**Point 4:** *L73-L76: A discussion of the model results would be inappropriate here.*

**Response:** It is really true as Reviewer suggested that this discussion are not appropriate here. After careful consideration, we delete this part of the content.

**Point 5:** *L76-L80: This is the purpose of your research, please state it on a separate line. Add your content chapter arrangement in this part.*

**Response:** We changed the position of the research purpose, and the chapter arrangement was placed after this paragraph.

**Point 6:** *L134-L139: The expression is fragmented and not well connected to the literature. Please integrate this section with the "contribution" in the introduction section and highlight your innovation in this section.*

**Response:** Thank you for your valuable comments. We reorganized this section and highlighted the contribution and innovation of our research.

**Point 7:** *L239: "MGWR uses different bandwidths for each variable, unlike the classical GWR model, which requires different levels of spatial smoothing for each variable;" I think what you are trying to convey is that MGWR can “requires different levels of spatial smoothing for each variable”, but the subject of the second half of this sentence is GWR. Please verify if the meaning of this sentence matches what you are trying to express.*

**Response:** Thank you very much for your valuable comments. We are sorry for the mistake due to our negligence. The correct meaning of this sentence is as you point out. We have corrected the error in this sentence.

**Point 8:** *It is best not to have a figure in the discussion section about the results. Please put Figure 5 and related contents in another appropriate place. It is suggested that Chapter 3 be discussed in several subsections, and the discussion on Figure 5 could be a subsection of Chapter 3.*

**Response:** We put Figure 5 in Chapter 4 and divided Chapter 4 into different sections depending on the content.

**Point 9:** *! Please be responsible for your own paper, read it carefully and correct all details of the mistakes.*

*(1) L46, L318: A period is missing at the end of the sentence.*

*(2) The content of the form should be standardized: Note that the number of decimal points in the tables is the same for the same category of data, so that it doesn't look cluttered.*

*(3) Make sure that the first letter of the same content is the same in different tables.*

*(4) Please unify the citation of all references, such as L215, etc.*

*(5) L226-L228: The letters of the formula and the explanation should correspond in case. j is a lowercase letter.*

*(6) Please check the text for all grammatical errors. E.g. L267: S1 and S2 Tables show …..; L271: shows?*

**Response:** We are very sorry for neglecting these details in the whole paper. We read the manuscript carefully and correct it for detail mistakes.

**Point 10:** *L123: “Second,…..”*

**Response:** Thank you for your kind comments. We have changed the sentence.

**Point 11:** *L149: Two “study area” in one sentence is wordy. The former emphasizes the object of study and the latter emphasizes the area.*

**Response:** To reduce wordy, we rewrote the sentence according to the context.

**Point 12:** *It is not necessary to emphasize "in this paper” or “in this study". E.g. L163 and L166.*

**Response:** We carefully checked the full manuscript and reduced the number of words "in this paper" and "in this study".

**Point 13:** *L168: too many “and”*

**Response:** Thank you for your kind comments. We rewrote the sentence to avoid too many “and”.

**Point 14:** *Use "(1) Variable 1." or other tag before each variable explanation so that it corresponds to your variables selections in the first paragraph.*

**Response:** Thank you for your valuable comments. We added some variable labels like " **(1) POIs** " before each category of variable explanation.

**Point 15:** *L235-L237: It should be placed before table1.*

**Response:** We changed the position of this section and placed before Table 1.

**Point 16:** *In order to make it easier for readers to distinguish, I suggest writing “Morning peak 1 model (pick-ups), Morning peak 2 model (drop-offs) ’’in Table 2, so as in Table 3-5.*

**Response:** Based on your valuable comments, we have changed the corresponding contents of Tables 2 to 5.

We tried our best to improve the manuscript and made some changes in the manuscript. These changes will not influence the main content and framework of the paper. And here we did not list the changes but marked in red and blue in revised paper.We appreciate for your warm work earnestly, and hope that the correction will meet with approval.Once again, thank you very much for your comments and suggestions.
