# Supplementary material for: Impact of built environment on residential online car-hailing trips: Based on MGWR model
Source: PLoS One. 2022 Nov 17;17(11):e0277776. doi: 10.1371/journal.pone.0277776 (PMC9671434; doi:10.1371/journal.pone.0277776)
Supplement: Attachment — Submitted filename: Response to Reviewers.docx [file pone.0277776.s006.docx]

Dear Editors and Reviewers:

Thank you for your letter and for the reviewers’ comments concerning our manuscript entitled “Impact of built environment on residential online car-hailing trips: based on MGWR model” (ID: PONE-D-22-20651R2). Those comments are all valuable and very helpful for revising and improving our paper, as well as the important guiding significance to our researches. We have studied comments carefully and have made correction which we hope meet with approval. Revised portion are marked in red in the paper. The main corrections in the paper and the responds to the reviewer’s comments are as fllowing.

We sincerely hope the new version meet with the publication standard of plos one. Please let us know if anything else is needed and we look forward to hearing from you soon.Thank you and best regards.

Yours sincerely,

Yan Cao

Corresponding Author:

Yongzhong Tian

Associate Professor

School of Geographical Sciences, Southwest University

Chongqing 400715, China

Tel/Fax: +86-17729619166

Email: tyzlf@swu.edu.cn

**Responds to the reviewer #1’s comments**

**Point 1:** *L62 and L63 are suggested in the same paragraph*

*L115: two periods at the end of the sentence.*

*L115 and L116 should be in the same paragraph*

*L248: S1 and S2 show*

*L334: Table 5, there's an extra vertical line to the right of the table*

*Others…*

**Response**: Thank you for your valuable comments. We are sorry for the mistake due to our negligence. First, as you suggested, we combined L62 and L63, L115 and L116 into the same paragraph, respectively. Second, We corrected errors in L115, L248 and L334. In addition, we have read the full manuscript again carefully and corrected the detail errors, especially the use of Spaces between words.

We tried our best to improve the manuscript and made some changes in the manuscript. These changes will not influence the main content and framework of the paper. And here we did not list the changes but marked in red in revised paper. We appreciate for your warm work earnestly, and hope that the correction will meet with approval. Once again, thank you very much for your comments and suggestions.
